# Editorial: Novel therapeutic strategy against obesity by targeting thermogenic fat

**DOI:** 10.3389/fendo.2022.1052966

**Published:** 2022-10-14

**Authors:** Ruping Pan, Takeshi Yoneshiro, Yutaka Hasegawa, Xinran Ma, Yong Chen

**Affiliations:** ^1^ Department of nuclear medicine, Tongji Hospital, Tongji Medical College, Huazhong University of Science and Technology, Wuhan, China; ^2^ Department of Nutrition, School of Nursing and Nutrition, Tenshi Collage, Tokyo, Japan; ^3^ Division of Metabolic Medicine, Research Center for Advanced Science and Technology, The University of Tokyo, Tokyo, Japan; ^4^ Division of Diabetes, Metabolism and Endocrinology, Department of Internal Medicine, Iwate Medical University, Yahaba, Japan; ^5^ Shanghai Key Laboratory of Regulatory Biology, Institute of Biomedical Sciences, School of Life Sciences, East China Normal University, Shanghai, China; ^6^ Department of Endocrinology and Metabolism, Fengxian Central Hospital Affiliated to Southern Medical University, Shanghai, China; ^7^ Shanghai Frontiers Science Center of Genome Editing and Cell Therapy, Shanghai Key Laboratory of Regulatory Biology and School of Life Sciences, East China Normal University, Shanghai, China; ^8^ Department of Endocrinology, Internal Medicine, Tongji Hospital, Tongji Medical College, Huazhong University of Science and Technology, Wuhan, China; ^9^ Branch of National Clinical Research Center for Metabolic Diseases, Hubei, China; ^10^ Laboratory of Endocrinology and Metabolism, Tongji Hospital, Tongji Medical College, Huazhong University of Science and Technology, Wuhan, China

**Keywords:** obesity, brown adipose tissue, beige adipose tissue, thermogenic mechanism, clinical application

## Introduction

With the development of world economy, changes in dietary pattern and life pattern result in a rapid rise of obesity in population worldwide. Adiposity causes the development of a series of metabolic diseases. Brown and beige adipose tissues have been discovered to be involved in the regulation of non-shivering thermogenesis, burning fat and generating heat, which are thus termed thermogenic adipose tissue. In particular, beige adipose tissue is special due to its arising in the white adipose tissue depot upon certain stimulation. Several receptor agonists binding to classic adrenergic receptors or G-coupled protein receptors have been shown to induce thermogenic fat activation and subsequently increase the energy expenditure in humans (1–3). Besides, various mechanisms are involved in the activation of thermogenic adipose tissue, especially, the beiging process is heterogenous (4). Scientists are still making great efforts to discover potential factors involved in this process that could be targeted to combat obesity but with least side effects. However, big challenges, particularly a transition from animal experiments to clinical practices, still remain, which require further efforts. This Research Topic reviews novel and newly discovered regulatory mechanisms involved in thermogenic adipose tissue physiology, new viewpoints, current advancements and remaining challenges in this field for a better understanding of human thermogenic adipose tissue physiology and discovery of promising therapeutic strategies to combat obesity by targeting human thermogenic adipose tissues.

## Human brown adipose tissue

Regardless of its composition (brown or beige), thermogenic adipose tissues in humans have been visually shown by means of ^18^F-fluorodeoxyglucose positron emission tomography/computed tomography (^18^F FDG-PET/CT) due to an increased uptake of glucose upon adipose tissue activation (5). Early human studies show that cold exposure induces the glucose uptake in human BAT. Besides, BAT activity in humans is negatively correlated to age and BMI (5). Possibly, lower BAT activation in obese people is related to an impaired adrenergic signaling and lower expression of thermogenic genes in their WAT. However, on the other hand, the quality of BAT in obese subjects may also be concealed by certain circumstances. In this regard, Kulterer et al has discovered that the volume and activity of BAT is similar between lean and obese populations, though the frequency of BAT activation is lower in obese individuals. In particular, visceral adipose tissue mass but not the whole-body fat mass or subcutaneous WAT is inversely correlated to BAT activity in both lean and obese people. Thus, it is suggested that glucose uptake in human BAT could not incisively be the only evaluation of BAT activity, especially between lean and obese individuals, since obese individuals may present an insulin resistance and are less sensitive to ^18^F FDG per se. Other radionuclide imaging agents such as ^13^C-acetate or fatty acid tracers could be used to estimate the oxidative capacity and fatty acid synthesis in human BAT. Furthermore, Unlike the consistent view, Monfort-Pires et al have found that there is no association between BAT activity/volume and adiposity in lean and obese populations living in tropical areas. Instead, a strong positive correlation is found between BAT triglyceride content and visceral adiposity as well as cardiovascular risk markers in not only lean but also overweight/obese individuals, suggesting that BAT triglyceride content could be a potential biomarker for the evaluation of cardiometabolic profiles. Besides, certain BAT related characteristics in tropical areas is inconsistent with those in temperate climate regions, suggesting possible BAT whitening in warm acclimation. Thus, the new insights of these articles provide new viewpoints for learning human BAT in the future.

## Thermogenic mechanisms and advancements

Knowledges on thermogenic mechanisms are mostly contributed from rodents. Human BAT maybe composed of both brown and beige adipocytes. The uneven activity/volume of BAT in independent individuals could be attributed to mechanisms including WAT beiging and BAT whitening, since evidence show that human infant has classic BAT located in the interscapular region. According to previous findings, the development of beige adipose tissue is heterogenous and complicated. Except for classic signaling pathway involved b3-adrengic receptor activation and uncoupled protein 1 (UCP1)-regulated oxidative phosphorylation in mitochondria, Wang et al. also summarized several adaptive thermogenic mechanisms, which are UCP1-independent as well as several novel regulatory mechanisms of beiging which are new to this field. Furthermore, Pan and Chen reviewed advancements on the receptor agonists development and other prominent mechanisms (some receptor signaling pathways), which are involved in the regulation of human BAT physiology, though most of them are based on findings in rodents. Of note, several receptor agonists have been shown their therapeutic potentials in maintaining metabolic balance in humans, such as adrenergic receptor and adenosine receptor agonists, as reviewed by Pan and Chen however, research on this field still requires further efforts.

Wei and Shi has systematically reviewed the function of Rho kinases (ROCKs) in the regulation of metabolic equilibrium in major metabolic organs (liver, brain, skeletal muscle and adipose tissue), focusing on their isoform-specific roles in the regulation of white/beige adipogenesis and thermogenesis. They also summarized miRNAs that regulate RhoA/ROCK signaling. Their work provides a more comprehensive knowledge of the roles of RhoA/ROCK signaling in metabolic balance and suggests ROCK inhibitors could potentially be used for the treatment of obesity and related metabolic disorders.

## Respectives

Targeting brown and beige adipose tissues is a potential therapeutic strategy to combat obesity due to its competent capacity on fat burning. Human studies are very limited. It is of vital significance to transmit the knowledges from experiments to clinical practices and improve the clinical applications of anti-obesity therapeutics. The original and review articles published in this Research Topic provide new insights on human BAT biology and trends on the treatment of obesity and its related metabolic abnormalities by targeting thermogenic adipose tissues.

## Author contributions

YC wrote the manuscript. RP, TY, YH and XM edited the manuscript. All authors contributed to the article and approved the submitted version.

## Funding

This work was supported by a grant from National Natural Science Foundation of China (grant number 82070859 and 82270910 to YC and RP) and a grant from Tongji Hospital in HuaZhong University of Science and Technology (grant number 2201103295 to YC).

## Acknowledgments

We are grateful to all the authors and reviewers for their contributions to this Research Topic.

## Conflict of interest

The authors declare that the research was conducted in the absence of any commercial or financial relationships that could be construed as a potential conflict of interest.

## Publisher’s note

All claims expressed in this article are solely those of the authors and do not necessarily represent those of their affiliated organizations, or those of the publisher, the editors and the reviewers. Any product that may be evaluated in this article, or claim that may be made by its manufacturer, is not guaranteed or endorsed by the publisher.
